# Associated diseases and their effects on disease course in patients with chronic non-bacterial osteomyelitis: retrospective experience from a single center

**DOI:** 10.1007/s10067-025-07306-1

**Published:** 2025-01-14

**Authors:** Betül Öksüz Aydın, Fatma Aydın, Özen Taş, Onur Bahçeci, Zeynep Birsin Özçakar

**Affiliations:** 1 Division of Pediatric Rheumatology, Department of Pediatrics, Ankara University Faculty of Medicine, Ankara, Turkey; 2 Division of Pediatric Rheumatology and Nephrology, Department of Pediatrics, Ankara University Faculty of Medicine, Ankara, Turkey

**Keywords:** Autoinflammatory, Children, Chronic non-bacterial osteomyelitis (CNO), Comorbidities

## Abstract

**Objective:**

Chronic non-bacterial osteomyelitis (CNO) is a rare autoinflammatory bone disease associated with other chronic inflammatory diseases such as familial Mediterranean fever (FMF), juvenile idiopathic arthritis (JIA), spondylarthropathies, inflammatory bowel disease (IBD), and pyoderma gangrenosum. We aimed to describe the clinical and follow-up characteristics of patients with CNO and to compare findings between patients with and without comorbidities.

**Methods:**

The clinical records of patients with CNO who were followed up in our pediatric rheumatology clinic between 2018 and 2023 were reviewed. Patients were divided into two groups according to the presence or absence of comorbidities. The clinical, laboratory, and radiological characteristics and treatments of the groups were compared.

**Results:**

The study included 40 patients (65% male) diagnosed with CNO. The median (IQR) age at symptom onset was 10 (6.4) and at diagnosis was 11.5 (5.9) years. Fourteen (35%) patients had comorbidities. The comorbidities were FMF (*n* = 9), IBD (*n* = 3), uveitis (*n* = 3), psoriasis (*n* = 1) and acne conglabatae (*n* = 2). The group with comorbidities had higher number of bones involved (3 or more bones) (78.6% versus 42.3%) (*p* = 0.028), and 78.6% of patients with comorbidities received biologic treatment, while only 23.1% of patients without comorbidities were treated with biologics (*p* = 0.001).

**Conclusion:**

Familial Mediterranean fever, uveitis, IBD, psoriasis and acne conglabata were found to be the clinical conditions associated with CNO. Patients with CNO who had comorbidities appeared to have a more severe phenotype of the disease accompanied with more bone involvement and requiring more biologic treatment.

**Table:** 

**Key Points** • *Chronic non-bacterial osteomyelitis (CNO), an auto-inflammatory bone disease, can be seen in association with other inflammatory conditions such as familial Mediterranean fever (FMF), uveitis, inflammatory bowel disease (IBD), psoriasis, and acne conglobata.* • *If CNO is associated with another inflammatory disease, the number of bones involved may be higher and patients may need more intensive treatments, such as biologics.* • *CNO may coexist with one or more inflammatory diseases, which may exacerbate the disease phenotype.*

## Introduction

Chronic non-bacterial osteomyelitis (CNO) is a rare autoinflammatory bone disease with a wide clinical spectrum from unifocal to multifocal lesions. CNO was first described in 1972 by Giedion et al. [1]. The average age at diagnosis is 10 years, and it is two times more common in girls than in boys [2]. Overproduction of proinflammatory cytokines (IL-1β, IL-6, TNF-α) and decreased production of anti-inflammatory cytokines (IL-9, IL-10, IL-18) have been implicated in the pathogenesis. It is thought that the increased osteoclast activity resulting from the imbalance in cytokine production is responsible for the bone findings. The identification of affected individuals in first-degree relatives supports the genetic predisposition in these patients [3].

Clinical findings include insidious, recurrent bone pain in one or more regions [4]. The metaphysis of long bones is most commonly involved. Systemic symptoms such as fever, fatigue, and malaise occur in about 17–20% of patients. Extraosseous clinical manifestations include cutaneous, gastrointestinal, and rarely ocular and cardiac involvement and are seen in more than one-third of patients [5].

Inflammatory markers may be moderately to severely elevated. A variety of radiological imaging techniques can be used for diagnosis, but whole-body magnetic resonance imaging (WB-MRI) remains the gold standard. First-line treatment is non-steroidal anti-inflammatory drugs (NSAIDs), particularly effective in CNO with peripheral involvement. Disease-modifying drugs (sulfasalazine, methotrexate), TNF-α blockers, and bisphosphonates may be used if bone pain or systemic inflammation persists despite NSAID treatment.

Studies have shown that other chronic inflammatory diseases such as juvenile idiopathic arthritis (JIA), spondylarthropathies, inflammatory bowel disease (IBD), pyoderma gangrenosum, and SAPHO syndrome (synovitis, acne, pustulosis, hyperostosis, osteitis) are associated with CNO in about 20% of cases [4]. As CNO is a rare disease, the definition of the associated diseases will help to better understand the pathogenesis and course of the disease and to better define treatment approaches. The aim of this study was to present the clinical and follow-up characteristics of patients with CNO and to compare these between patients with and without coexisting conditions.

## Methods and materials

The patients with CNO who were followed up between 2018 and 2023 were included in the study. The diagnosis of CNO was made based on clinical and imaging findings after excluding any infectious or malignant diseases according to Jansson et al. [6]. Only patients with a minimum follow-up duration of 6 months were included in this study. Patients with inadequate follow-up or incomplete data were excluded from the study.

Demographic characteristics, clinical course, laboratory and imaging findings, treatments, and comorbidities were recorded. Magnetic resonance imaging results were evaluated by experienced pediatric radiologists. Patients had to be evaluated with a WB-MRI comprised of a STIR sequence of a coronal view of the whole body, a sagittal view of the entire spine and ankles, and an axial view of the pelvis and knees.

Clinical remission was defined as the absence of pain complaints in patients under treatment for at least 6 months of follow-up. Radiologic remission was defined as the disappearance of previous lesions with no new lesions on follow-up MRI within the first year of treatment.

The patients were classified into two groups based on the presence or absence of comorbidities. Clinical, laboratory, and radiologic characteristics of the groups were compared. Informed consent was obtained from the patients and parents. This study was approved by the ethics committee of our hospital (reference number: 2024/94).

## Statistical analysis

The statistical analyses were conducted using the software SPSS version 11.5. The normality of variables was tested using visual (histograms, probability plots) and analytical methods (Kolmogorov–Smirnov test). Descriptive statistics were presented as mean ± standard deviation for quantitative variables with normal distribution, median (IQR) for variables with non-normal distribution, and percentage for qualitative variables. Mann–Whitney *U* test was used to compare the means of two independent groups. The relationship between qualitative variables was evaluated by chi-square test or Fisher’s exact test. Statistical significance was defined as *p* < 0.05.

## Results

The study included 40 patients (*n* = 26, 65% male) diagnosed with CNO. The median (IQR) age at symptom onset was 10 (6.4) and at diagnosis was 11.5 (5.9) years. The most common presenting complaints were joint pain (*n* = 31, 77.5%) and bone pain (*n* = 31, 77.5%). The most common physical examination finding was pain on bone palpation (*n* = 10, 25%).

Thirty-four (85%) patients were diagnosed with WB-MRI, 3 with regional MRI, and 3 with regional MRI and scintigraphy. Multifocal involvement was present in 38 (95%) patients, and unifocal involvement was present in only two patients in the clavicle and humerus. Four patients (10%) had arthritis. Only the upper extremities were involved in 3 (7.5%), only lower extremities in 23 (57.5%), and both upper and lower extremities in 14 (35%) patients.

The most common MRI finding was bone marrow edema (*n* = 37, 92.5%). Other MRI findings included joint effusion in 16 patients (40%), osteitis in 7 patients (17.5%), and thickening of the synovium in 2 patients. 8 patients (20%) had sacroiliitis.

The median (IQR) white blood cell count at the time of diagnosis was 8065/mm^3^ (3438), C-reactive protein (CRP) was 9.2 mg/L (16.7), and erythrocyte sedimentation rate (ESR) was 18.5 (22.5) mm/h. Elevated acute phase reactants (APR) in at least one of the ESR or CRP values were present in 27 patients (67.5%).

Of our patients, 47.5% had consanguinity history and 42.5% had a family history of rheumatological disease. Antinuclear antibody (ANA) was positive in 9 patients (22.5%). Two patients (5%) were HLA-B27 positive. Eleven patients (27.5%) had *MEFV* mutations (*3 M694V heterozygote, 2 M694V homozygote, 2 M694V/V726A, 1 M680I/V726A, 1 E148Q heterozygote, 1 E148Q homozygote, 1 M680I heterozygote mutation*).

All demographic, clinical, radiological findings, treatments, and prognoses of the patients are shown in Table 1.

**Table 1:** Demographic, clinical, and radiological findings and prognosis of the patients

Demographic and clinical characteristics	*n* = 40 (%) Median (IQR)
Gender	
Male	26 (65)
Age at diagnosis (year)	11.5 (5.9)
Age of symptoms onset (year)	10 (6.4)
Patients with comorbidities	14 (35)
Bones involved in MRI	
Bones adjacent to sacroiliac joint	21 (52.5)
Femur	16 (40)
Vertebra	13 (32.5)
Tibia	12 (30)
Tarsal bones	12 (30)
Multifocal	38 (95)
Unifocal	2 (5)
ANA positivity	9 (22.5)
HLA-B27 positivity	2 (5)
MEFV mutation	11 (27.5)
Elevated acute phase reactants	27 (67.5)
ESR at diagnosis (mm/h)	18.5 (22.5)
CRP at diagnosis (mg/L)	9.2 (16.7)
NSAIDs use	40 (100)
cDMARD use	17 (42.5)
bDMARD use	17 (42.5)
Clinical remission	35 (87.5)
Remission with treatment	31 (77.5)
Remission without treatment	4 (10)
Radiological remission (*n* = 30)	17 (56.7)

*MRI* magnetic resonance imaging, *ANA* antinuclear antibody, *MEFV* *MEditerranean FeVer*,* ESR* erythrocyte sedimentation rate, *CRP* C-reactive protein, *NSAIDs* non-steroidal anti-inflammatory drugs, *cDMARD* conventional disease-modifying antirheumatic drugs, *bDMARD* biologic disease-modifying antirheumatic drugs. ^*^Bold font indicates statistical significance

The median (IQR) follow-up period was 37.5 (45.5) months. All patients used NSAIDs, and eight (20%) of them were treated only with NSAIDs. Seventeen patients (42.5%) received MTX, and 8 (47%) of them had remission with MTX. The median (IQR) treatment duration of patients treated with corticosteroids (*n* = 10, 25%) was 3 (3) months. Four patients (10%) received bisphosphonates, and 2 out of 4 had vertebral lesions with low BMD for age. One patient had low BMD for age with other bone involvements. One patient had been started on bisphosphonate treatment at another center due to NSAID resistance. Seventeen patients (42.5%) received biologic agents (anti TNF). Thirteen patients (32.5%) were treated with adalimumab. One patient was switched to infliximab while on adalimumab due to the persistence of uveitis, then switched back to adalimumab due to allergic side effects. Four patients (10%) were treated with etanercept, and one patient was switched from etanercept to adalimumab due to inadequate response. Detailed information about therapeutic agents are given in Table 2.

**Table 2:** Treatments used, their duration, and effectiveness

Drugs	Patients receiving this treatment *n* (%)	Complete clinical response in patients with CNO *n* (%)	Patients with comorbidities receiving this treatment *n* (%)	Complete clinical response in patients with comorbidities *n* (%)	Treatment duration (months) median (IQR) (min–max)
NSAIDs	40 (100)	8 (20)	14 (100)	1 (7.1)	24 (22.5)
MTX	17 (42.5)	8 (47)	6 (42.8)	2 (33.3)	12.75 (7)
Salozoprine	3 (7.5)	1 (33.3)	1 (7.1)	–	18 (15–21)
Bisphosphonate	4 (10)	1 (25)	4 (28.6)	1 (25)	**–**
Adalimumab	13 (32.5)	11 (84)	8 (57.1)	8 (100)	21 (22)
Etanercept	4 (10)	2 (50)	2 (14.3)	2 (100)	35.5 (45.5)
Infliximab	2 (5)	1 (50)	2 (14.3)	2 (100)	6.5 (5–8)

*NSAIDs* non-steroidal anti-inflammatory drugs, *MTX* methotrexate, *CNO* chronic non-bacterial osteomyelitis

Thirty-five (87.5%) of the patients were clinically in remission when they were included in the study. While 31 patients (77.5%) were in remission with treatment, 4 patients (10%) were in remission without treatment. Clinical remission was not achieved in 5 patients. Ten (25%) patients developed relapse after a median (IQR) of 10.5 (12.5) months of treatment discontinuation.

Control MRI was performed in 30 patients in the first year of treatment. Thirteen (43.3%) of 30 patients had radiologically new lesions in addition to resolved old lesions detected on control MRI. Seventeen patients (56.7%) had regressed existing lesions on control MRI and were considered to be in radiologic remission.

During follow-up, vertebral height loss developed in 3 patients (7.5%), osteopenia in 3 patients (7.5%), and sclerosis in 2 (5%). There were 4 patients (10%) with a body weight below 3 percentile before treatment and no patient with a body weight below 3 percentile after treatment. There were 3 patients (7.5%) with height below 3 percentile before treatment and 1 patient (2.7%) with height below 3 percentile after treatment.

Fourteen (35%) patients had comorbidities. The comorbidities were familial Mediterranean fever (FMF) (*n* = 9), IBD (*n* = 3), uveitis (*n* = 3), psoriasis (*n* = 1), and acne conglabatae (*n* = 2). Two of the IBD were ulcerative colitis and one was Crohn’s disease. Among patients with comorbidities, one had both FMF and uveitis, another had FMF and acne conglabatae, and one had FMF and IBD. Uveitis was also present in the patient with psoriasis. Of the 3 patients with uveitis, two had intermediate uveitis and one had panuveitis, all of them had blurred vision and uveitis findings were severe.

When we compared the patients with and without comorbidities, it was found that 92.9% of patients with comorbidities were male, which was statistically significant (*p* = 0.013). Additionally, the group with comorbidities had a higher family history of rheumatologic disease (57.1% versus 34.6%), but this result was not statistically significant (*p* = 0.169). There were no statistically significant differences between the two groups in terms of other demographic characteristics. When the accompanying symptoms were compared between the groups, bone pain was statistically significantly (*p* = 0.044) more common in patients without comorbidity.

The mean ± SD number of bone involvement was 2.9 ± 1.41. The group with comorbidities had the highest number of involved bones (3 or more bones) (*n* = 11, 78.6%), and this result was statistically significant (*p* = 0.028).

In terms of MRI findings, joint effusion (50% versus 34.6%) and sacroiliitis (21.4% versus 19.2%) were more common in the group with comorbidities than without comorbidities, but these results were not statistically significant (*p* values *p* = 0.343, *p* = 1, respectively).

Of the 13 patients with radiologically new lesions detected on post-treatment control MRI, 10 were in the group without comorbidities, and this result was not statistically significant (*p* = 0.36). Relapse developed after a mean of 2.3 months in those with comorbidities, while relapse developed after a mean of 4.2 months in the group without comorbidities (*p* = 0.9). Of the 8 patients who developed complications, 5 (35.7%) had a comorbidity (*p* = 0.102).

A comparison of groups with and without comorbidities is given in Table 3.

**Table 3:** Comparison of groups with and without comorbidities

	Patients with comorbidities ***n*** = 14 (%) Median (IQR)	Patients without comorbidities ***n*** = 26 (%) Median (IQR)	***p***value
Gender			
Female	1 (7.1)	13 (50)	**0.013**
Male	13 (92.9)	13 (50)	
Age of symptoms onset (year)	7.7	9.8	0.11
Age at diagnosis (year)	10.5	11.5	0.44
Symptoms			
Bone pain	8 (57.1)	23 (88.5)	**0.044**
Night pain	6 (42.9)	5 (19.2)	0.147
Morning stiffness	7 (50)	9 (34.6)	0.343
Bones Involved in MRI			
Bones adjacent to sacroiliac joint	8 (57.1)	13 (50)	0.666
Femur	7 (50)	9 (34.6)	0.343
Vertebrae	5 (35.7)	8 (30.8)	1
Tibia	4 (28.6)	8 (30.8)	1
Tarsal bones	6 (42.9)	6 (23.1)	0.234
Number of involved bones (3 or more bones)	11 (78.6)	11 (42.3)	**0.028**
ANA positivity	4 (28.6)	5 (19.2)	0.694
Elevated acute phase reactants	10 (71.4)	17 (65.4)	1
ESR at diagnosis (mm/h)	15.5 (30.8)	19 (17.5)	0.887
CRP at diagnosis (mg/L)	11.8 (26.4)	6.15 (12.7)	0.100
cDMARD use	6 (42.9)	11 (42.3)	0.973
bDMARD use	11 (78.6)	6 (23.1)	**0.001**

*MRI* magnetic resonance imaging, *ANA* antinuclear antibody, *ESR* erythrocyte sedimentation rate, *CRP* C-reactive protein, *cDMARD* conventional disease-modifying antirheumatic drugs, *bDMARD* biologic disease-modifying antirheumatic drugs. ^*^Bold font indicates statistical significance

There was no significant difference between the two groups in terms of NSAIDs, conventional disease-modifying antirheumatic drugs (cDMARDs), steroid, and bisphosphonate usage. Of the patients with comorbid diseases, 78.6% received biologic disease-modifying antirheumatic drugs (bDMARDs), while only 23.1% of patients without comorbidities received them and this result was statistically significant (*p* = 0.001).

Among patients with comorbidities, one of the three IBD patients was started on infliximab after evaluation by the pediatric gastroenterology department for the patient’s existing IBD; one patient with FMF and IBD was given adalimumab due to CNO clinic, and the other patient was switched from NSAID to adalimumab due to persistent pain and the presence of new lesions on MRI. All three patients with severe uveitis received corticosteroids, MTX, and adalimumab. Two patients with acne conglabata were treated with adalimumab for skin lesions. Only two out of nine FMF patients had CNO under control with NSAID treatment, while the other 4 patients with FMF and CNO required MTX and/or anti-TNF treatment. Details about biological agents are given in Table 2.

## Discussion

In this study, we found that 35% of pediatric patients with CNO had comorbidities, including FMF, uveitis, IBD, psoriasis, and acne conglobata. Morever, we observed an approximately twofold increase in the number of bone lesions and an increased need for biologic treatment in patients with comorbidities. Accordingly, CNO patients with comorbidities seemed to have a more severe disease phenotype.

It is known that among patients with CNO, girls are affected approximately 2–4 times more frequently than boys [7]. In contrast to European publications, our study did not show female predominance, similar to previous publications from our country [8–12]. However, interestingly, we found that the rate of males with co-morbidities was significantly higher. Of our patients, 47.5% had consanguinity history and 42.5% had a family history of rheumatological disease. Although the genetics of CNO has not been explained, the clustering of the disease in some families and its association with other inflammatory diseases such as IBD, palmoplantar pustulosis, and psoriasis suggest a genetic basis for CNO [13].

Familial Mediterranean fever is the most common autoinflammatory disease characterized by recurrent episodes of fever and serositis lasting 1–3 days [14]. Increased frequency of various diseases such as Behçet’s disease, Henoch-Schönlein purpura, IBD, psoriasis, and JIA has been reported in patients with FMF [15–18]. In FMF, the gene responsible, *MEditerranean FeVer *(*MEFV*), is on chromosome 16p and encodes a protein called pyrin. The mutated pyrin interacts with the inflammasome, activating caspase-1 and causing excessive secretion of IL-1β and other cytokines, including IL-6 and IL-18. Mutated pyrin may explain the association between inflammatory mediators, FMF, and associated comorbid autoinflammatory diseases [19].

The coexistence of CNO and FMF was first reported in 2010 by Shimizu et al. Over time, similar case series describing the coexistence of FMF and CNO have been reported [20]. The prevalence of FMF in CNO patients is unknown. In our study, we found that FMF was the most common comorbidity with a rate of 22.5%. Avar-Aydın et al. reported that 5 out of 18 patients with CNO (27.8%) were found to have at least one exon 10 mutation, and these patients with *MEFV* mutations had an earlier onset of CNO, higher APR, lower hemoglobin concentrations, and a higher number of bone lesions at onset [9]. These results support that CNO patients with *MEFV* mutations were more likely to have a persistent course of the disease. In patients with CNO, periodic fever syndromes should be considered when recurrent fever, abdominal pain, or unexplained APR elevation develops, especially in FMF endemic areas. On the other hand, FMF patients with persistent musculoskeletal symptoms should be evaluated for CNO.

Inflammatory bowel disease presents with gastrointestinal symptoms such as abdominal pain, diarrhea, and weight loss, but may also present with extraintestinal symptoms such as arthralgia, arthritis, and sacroiliitis, which may be confused with symptoms of JIA or CNO. The association between IBD and CNO was first recognized by Kahn et al. [21]. Although the mechanisms associated with bone inflammation in CNO and intestinal inflammation in IBD are not fully elucidated, Zhao et al. suggested a possible link between the microbiome and CNO [22]. It is conceivable that disturbances in the balance of gut microorganisms may extend beyond the gut and lead to the development of bone inflammation in CNO. In patients with CNO, disruption of the intestinal barrier leads to inflammation and release of cytokines, which may play a role in the extraintestinal manifestations of IBD, particularly bone inflammation [23]. Studies have shown that IL-10 dysregulation is a common pathophysiological mechanism between CNO and IBD [24]. TNF-α upregulation has also been implicated in the pathogenesis of IBD and CNO, and a favorable response to anti-TNF-α may support this suggestion [25]. There is also evidence of a genetic predisposition to both CNO and IBD [24].

In our study, IBD was concomitant with CNO in 3 patients (7.5%), 2 with ulcerative colitis and 1 with Crohn’s disease. Dushnicky et al. analyzed 600 patients with IBD and 47 with chronic recurrent multifocal osteomyelitis (CRMO) and found that 7 pediatric patients were diagnosed with both IBD and CRMO [25]. This means that 14.8% of CNO patients were associated with IBD. This was about 2 times higher than in our study. Otherwise, 1% of pediatric patients with IBD also had CNO and most patients had an initial diagnosis of IBD and were later diagnosed with CNO. In the study by Ariadni Tzaneti et al. involving 40 patients, 21 (52.5%) of the patients were initially diagnosed with CNO, 14 (35%) were initially diagnosed with IBD, while 5 patients (12.5%) were diagnosed with concurrent CNO and IBD [26].

In patients with IBD with unexplained bone pain or joint complaints, the diagnosis of CNO should be kept in mind. Conversely, IBD should be considered in patients with CNO who present with chronic abdominal pain, diarrhea, or otherwise unexplained bloody stools.

Chronic non-bacterial osteomyelitis may be associated with cutaneous manifestations such as acne, psoriasis, palmoplantar pustulosis, and pyoderma gangrenosum [27]. Skin involvement is more common in SAPHO syndrome, which is considered the adult form of CNO. Although the pathogenesis of SAPHO syndrome is unclear, studies suggest that proinflammatory cytokines such as IL-8 and TNF-α may be triggered by Cutibacterium acnes infection and lead to skin lesions such as acne conglobata and a systemic inflammatory response [28]. Psoriasis has been reported in 4–22% of patients in previous studies and less than 10% in most studies [3, 29–31]. In our study, psoriasis was seen in one patient (2.5%). Acne conglobata, a severe form of nodular acne vulgaris, affects mainly adolescent boys [32]. In our study, acne conglobata was present in two patients, one of whom was a girl and the other a boy.

Pediatric-onset uveitis is rare and accounts for 5–10% of all uveitis cases [33]. Non-infectious uveitis can be autoimmune or autoinflammatory, and the most common causes of uveitis in this age group are idiopathic and JIA-related uveitis. In our study, uveitis was associated with CNO in 3 (7%) patients. Uveitis accompanying CNO is a rare condition. Among the diseases associated with CNO, uveitis has not been considerably reported. One of our patients with CNO had concurrent uveitis and psoriasis. Common cytokines in the etiologies of both psoriasis and uveitis may be thought to be effective in the formation of this condition. Two of our patients had intermediate uveitis, and one had panuveitis; all of them had blurred vision, and uveitis findings were severe. In this regard, it is important to know that uveitis may accompany CNO. Uveitis findings can be detected with ophthalmological examinations that may be performed at an earlier stage in patients diagnosed with CNO.

CNO is an autoinflammatory disease that primarily affects the bones, but it is known that diseases that develop on the basis of inflammation can be seen together. Two or sometimes three inflammatory diseases can coexist, but which one develops first varies. If the signs are inconsistent with the primary disease, it should be considered that a secondary disease may be present. Identifying the comorbid condition is very important both in terms of treatment management and disease prognosis.

In the study by Borzuztzky et al., patients with comorbid autoimmune diseases had more multifocal involvement and were a risk factor for treatment with immunosuppressive agents [5]. In our study, the number of involved bones was also higher in patients with comorbidities, and this may be the result of enhanced inflammation. Similar to the study by Cebecauerova et al., we observed that the need for biologic agents was more frequent in CNO patients with another inflammatory disease [34]. On the other hand, we detected that a higher number of new lesions developed on control MRIs of patients in those without comorbidities. This may result from more intensive treatment in patients with comorbidities. Complications were also more common (not significantly) in the comorbidity group, indicating that comorbidities can affect the prognosis of CNO adversely. In terms of MRI findings, joint effusion and sacroiliitis were more common but not significantly in the group with comorbidities. Joint findings may also be seen in CNO, but according to our findings, diseases that may accompany CNO should be kept in mind in the presence of joint findings.

The small number of patients and the retrospective nature may be limitations of our study. However, regarding rarity of CNO and associated conditions, such studies contribute greatly to the literature.

In conclusion, FMF, uveitis, IBD, psoriasis, and acne conglabata were found to be the clinical conditions associated with CNO. A significant finding was the increasing number of bone lesions and the need for more intensive treatment in those with comorbidities. Patients with CNO who had comorbidities appeared to have a more severe phenotype of the disease.

## Data Availability

The corresponding author can be contacted for this if necessary.
